# The effects of MAOA genotype, childhood trauma, and sex on trait and state-dependent aggression

**DOI:** 10.1002/brb3.96

**Published:** 2012-10-05

**Authors:** Floor E A Verhoeven, Linda Booij, Anne-Wil Kruijt, Hilâl Cerit, Niki Antypa, Willem Does

**Affiliations:** 1 Institute of Psychology, Leiden UniversityLeiden, The Netherlands; 2 Sainte-Justine Hospital Research CenterMontreal, Quebec, Canada; 3 Department of Psychiatry, University of MontrealMontreal, Quebec, Canada; 4 Department of Psychiatry, McGill UniversityMontreal, Quebec, Canada; 5 Leiden Institute of Brain and CognitionLeiden, The Netherlands; 6 Department of Psychiatry, Leiden University Medical CenterLeiden, The Netherlands

**Keywords:** Aggression, cognitive reactivity, depression, MAOA, sex, trauma

## Abstract

Monoamine oxidase A (MAOA) genotypic variation has been associated with variation in aggression, especially in interaction with childhood trauma or other early adverse events. Male carriers of the low-expressing variant (MAOA-L) with childhood trauma or other early adverse events seem to be more aggressive, whereas female carriers with the high-expressing variant (MAOA-H) with childhood trauma or other early adverse events may be more aggressive. We further investigated the effects of MAOA genotype and its interaction with sex and childhood trauma or other early adverse events on aggression in a young adult sample. We hypothesized that the association between genotype, childhood trauma, and aggression would be different for men and women. We also explored whether this association is different for dispositional (trait) aggression versus aggression in the context of dysphoric mood. In all, 432 Western European students (332 women, 100 men; mean age 20.2) were genotyped for the MAOA gene. They completed measures of childhood trauma, state and trait measures of aggression-related behaviors (STAXI), and cognitive reactivity to sad mood (LEIDS-R), including aggression reactivity. Women with the MAOA-H had higher aggression reactivity scores than women with the MAOA-L. This effect was not observed in men, although the nonsignificant findings in men may be a result of low power. Effects on the STAXI were not observed, nor were there gene by environment interactions on any of the aggression measures. A protective effect of the low-expression variant in women on aggression reactivity is consistent with previous observations in adolescent girls. In females, the MAOA-H may predispose to aggression-related problems during sad mood.

## Introduction

Monoamine oxidase A (MAOA) is an enzyme essential for the degradation of monoamines in the central nervous system (Oreland 1991). Previous research has shown that MAOA plays a major role in aggression. In one of the first studies, a point-mutation in the gene that codes for MAOA, causing complete MAOA deficiency, was associated with criminal and violent behaviors in males. This effect was seen over multiple generations in the family studied (Brunner et al. 1993). This link between lower MAOA enzyme activity and aggression has been confirmed in studies using animal models (Cases et al. 1995) and in human studies that used positron emission tomography to measure MAOA function in vivo (Alia-Klein et al. 2008; Soliman et al. 2011).

The MAOA gene is located on the X chromosome (Xp11.23-11.4) and has a variable number of tandem repeats (VNTR). Alleles with 3.5 or 4 copies lead to 2–10 times more efficient transcriptional activity (indicating high expression; MAOA-H) than alleles with three copies (low expression; MAOA-L) (Sabol et al. 1998).

An early study showed that maltreated boys with the MAOA-L genotype were at greater risk to develop antisocial problems than maltreated boys with the MAOA-H genotype (Caspi et al. 2002). This finding has been replicated (Foley et al. 2004; Huang et al. 2004; Kim-Cohen et al. 2006; Nilsson et al. 2006; Ducci et al. 2008; Cicchetti et al. 2010; Enoch et al. 2010) but not consistently (Young et al. 2006; Alia-Klein et al. 2008). Although most studies have shown that the MAOA-H variant is associated with less aggressive behavior in males, this variant may be a risk factor for increased aggressive behaviors in adolescent girls who experience early psychosocial risk factors (Sjöberg et al. 2007; Åslund et al. 2011).

Problems in aggression regulation are a common symptom of many psychiatric disorders. For instance, up to 30–40% of depressed patients seem to experience some form of aggression regulation problems during their depression, ranging from irritability (Perlis et al. 2009; Verhoeven et al. 2011) to anger attacks (Fava and Rosenbaum 1999; Van Praag 2001). Consistent with this, MAOA has been linked to both aggression and the development and pharmacological treatment of depression (Pare 1985; Aklillu et al. 2009). This may suggest that the relationship between MAOA and aggression depends on the context of aggression. Indeed, a previous study has shown that the effects of the MAOA gene on aggression are most prominent in an aggression-provoking situation (McDermott et al. 2009). It is therefore of interest to assess the role of the MAOA gene in aggression-related behaviors in the context of sad mood.

In this study, we investigated the effects of MAOA genotype and its interaction with sex and early adversity on different aspects of aggression in young adults, that is, state aggression but also aggression in the context of depression. We hypothesized that the association between genotype and childhood trauma would be different for men and women. Specifically, we expected that male carriers of the low-expression MAOA variant would express higher levels of aggression-related behaviors than carriers of the high-expression variant, in particular in the context of early adversity. We expected an opposite pattern in females.

## Methods

### Participants

A total of 432 healthy, nonsmoking participants aged between 18 and 35 participated in the study (332 women, 100 men). Participants were recruited via advertisements, flyers, and posters in the university buildings (University of Leiden, the Netherlands). Participants had to be of Western European descent (i.e., all four grandparents born in the Netherlands, Germany, France, Belgium, Luxemburg, Austria, Switzerland, Ireland, the United Kingdom, or Scandinavia). Exclusion criteria were medication use (including oral contraception) and a current depressive episode. The presence of more women than men in the current sample is useful because, unlike men, women can be either hetero- or homozygous for the MAOA genotype.

### Measures

Childhood trauma was measured using the 28-item version Childhood Trauma Questionnaire (CTQ) (Bernstein et al. 1997; Thombs et al. 2009). This self-report questionnaire has been validated both in clinical and in nonclinical samples. The CTQ has five subscales (Emotional abuse, Physical abuse, Sexual abuse, Emotional neglect, and Physical neglect) and each item is rated on a Likert scale ranging from 1 (never true) to 5 (very often true). We divided participants in two groups: those who reported none/minimal-to-moderate levels of childhood trauma and those who reported moderate-to-severe levels of childhood trauma. The distinction was based on severity norm scores from a sample of North American college students (Bernstein et al. 1997), with participants scoring lower than the cutoff score of 38 assigned to the none/minimal-to-moderate levels of childhood trauma group and those scoring over 38 assigned to the moderate-to-severe levels of childhood trauma group.

The Spielberger State-Trait Anger Expression Inventory (STAXI) (Van der Ploeg et al. 1982; Spielberger et al. 1983; Forgays et al. 1997) was used to measure aggression-related behaviors both as an emotional state and as a personality trait. Both versions of the STAXI consist of 10 items with a 4-point Likert scale.

Cognitive reactivity was measured with the Leiden Index of Depression Sensitivity – Revised (LEIDS-R) (Van der Does 2002, 2005; Williams et al. 2008). This 34-item self-report questionnaire has six subscales (Aggression Reactivity [AGG], Hopelessness/Suicidality Reactivity [HOP], Acceptance/Coping [ACC], Control/Perfectionism [CTL], Risk Aversion [RAV], and Rumination on Sadness [RUM]). It instructs participants to indicate how their thinking patterns change when they experience mild dysphoria. Questions are answered on a 0–4 Likert scale. The AGG and HOP subscales and the LEIDS-R total score were the primary outcome measures for this study.

The AGG and HOP reactivity subscales have been found to be strongly associated with irritability in depressed patients (Verhoeven et al. 2011) and with suicidality (Antypa et al. 2010; Verhoeven et al. 2011). The LEIDS-R total score is associated with serotonin vulnerability (response to tryptophan depletion) (Booij and Van der Does 2007) and with the interaction of serotonin transporter gene polymorphism and early-life events (Antypa and Van der Does 2010).

### Procedure

All measures were obtained in a single session. All participants signed informed consent prior to participation and either received €10 or study credits. The research was approved by the Ethics Committee of the Institute of Psychology of Leiden University.

Saliva samples were collected using Oragene Self-Collection Kits – DISC format (DNA Genotek Inc, Ottawa, Ontario, Canada); 200 μL of saliva was kept in lysis buffer (100 mmol/L NaCl, 10 mmol/L EDTA, 10 mmol/L Tris pH 8, 0.1 mg/mL proteinase K, and 0.5% w/v sodium dodecyl sulfate) until further processing.

#### DNA isolation

Genomic DNA was isolated from the samples using the Chemagic kit on a Chemagen Module I workstation (Chemagen Biopolymer-Technologie AG, Baesweiler, Germany). DNA concentrations were quantified by OD260 measurement and by agarose gel electrophoresis. The average yield was approximately 4 μg of genomic DNA per sample.

#### Polymerase chain reaction amplification

The region of interest from the MAOA gene was amplified by triplex polymerase chain reaction (PCR) using the following primers: a 6-carboxyfluorescein-labeled Medium Resolution (MR) primer (5′-GGATAACAATTTCACACAGG-3′), forward primer (5′-ggataacaatttcacacaggACAGCCTGACCGTGGAGAAG-3′), and a reverse primer (5′-GGACCTGGGCAGTTGTGC-3′). Typical PCR reactions contained between 10 and 100 ng genomic DNA template, 1 pmol of forward primer, and 10 pmol of labeled MR and reverse primers. PCR was carried out in the presence of 5% dimethyl sulfoxide with 0.3 U of BioThermAB polymerase (GeneCraft, Munster, Germany) in a total volume of 30 μL using the following cycling conditions: initial denaturation step of 5 min at 94°C, followed by 38 cycles of 30 sec 94°C, 30 sec 55°C, 30 sec 72°C, and a final extension step of 4 min 72°C.

#### Analysis of PCR products

One microliter of PCR product was mixed with LIZ-500 size standard and formamide and run on an AB 3100 genetic analyzer setup for genotyping with 50-cm capillaries. Results were analyzed using Genescan software version 3.7 (Applied Biosystems, Carlsbad, California) and alleles were scored visually.

### Statistical analyses

Following screening for accuracy of data entry and verification of statistical assumptions, chi-square statistics and generalized linear model (GLM) were used to investigate differences in demographic and trauma frequency between the genotypes. Next, the influence of genotype and its interactions with childhood trauma and sex were analyzed in two steps.

First, we included both men and women in the analyses, but excluded the heterozygotes (resulting in *N* = 276). In case of a significant sex by genotype interaction, a second set of analyses in female participants only was performed, including both homo- (HH and LL) and heterozygote (HL) females. Since MAOA genotype is X-linked, we chose to exclude men in this set of analyses to ensure we would only be looking at the effect of genotype without the effect of sex, while still obtaining sufficient power.

Data were analyzed using GLM for (M)ANOVA, including MAOA genotype, childhood trauma, and (when relevant) sex as a between-subject factor. In case of three-group comparisons Tukey's test was used. IBM SPSS 19 (IBM Corporation, Armonk, New York) was used for data analysis.

## Results

### Preliminary analyses

Genotype frequencies were as follows for men: L, 35%; H, 65%; and for women: LL, 11.10%; LH, 47%; HH, 41.90%. As the MAOA-LPR polymorphism is X-linked, the Hardy–Weinberg equilibrium can only be reported for women for whom frequencies were in the equilibrium (χ^2^(1) = 0.47; *P* > 0.05).

As data obtained on both STAXI scales were right-skewed, square root transformed values were used.

### Combined male/female sample (MAOA-H/HH vs. MAOA-L/LL)

Sample characteristics and mean scores on the behavioral measures, as a function of MAOA genotype, are presented in Tables 1 (women) and 2 (men)

**Table 1:** Sample characteristics women (*n* = 332)

	LL genotype (*n* = 37)	HL genotype (*n* = 156)	HH genotype (*n* = 139)
Age (mean ± SD)	19.6 ± 1.8	20.1 ± 3.01	20.0 ± 2.4
STAXI trait total score (mean ± SD)	16.3 ± 3.6	15.8 ± 3.7	17.1 ± 4.6
STAXI state total score (mean ± SD)	15.11 ± 3.8	14.5 ± 3.1	14.9 ± 3.5
LEIDS-R total score (mean ± SD)	37.6 ± 13.3	40.2 ± 15.7	42.1 ± 14.6
LEIDS-R HOP (mean ± SD)	4.3 ± 3.0	5.3 ± 4.0	5.1 ± 3.9
LEIDS-R ACC (mean ± SD)	1.8 ± 2.3	1.5 ± 1.8	1.5 ± 1.8
LEIDS-R AGG (mean ± SD)	5.8 ± 3.4	6.4 ± 4.4	7.1 ± 4.3
LEIDS-R CTL (mean ± SD)	7.6 ± 3.2	7.6 ± 3.9	7.7 ± 3.4
LEIDS-R RAV (mean ± SD)	8.6 ± 3.8	9.1 ± 3.9	9.7 ± 3.8
LEIDS-R RUM (mean ± SD)	9.6 ± 3.7	10.5 ± 4.1	11.0 ± 4.0
Past depression (MDQ) (% yes)	29.7	39.7	32.4
Abuse (% moderate to severe on CTQ)	16.2	13.5	20.1

STAXI, Spielberger State Trait Anger Expression Inventory; LEIDS-R, Leiden Index of Depression Sensitivity – Revised; HOP, Hopelessness; ACC, Acceptance; AGG, Aggression; CTL, Control; RAV, Risk Aversion; RUM, Rumination; MDQ, Mood Disorder Questionnaire; CTQ, Childhood Trauma Questionnaire.

**Table 2:** Sample characteristics men (*n* = 100)

	L genotype (*n* = 35)	H genotype (*n* = 65)
Age (mean ± SD)	20.6 ± 2.3	20.7 ± 3.1
STAXI trait total score (mean ± SD)	15.1 ± 4.2	15.4 ± 3.3
STAXI state total score (mean ± SD)	13.6 ± 2.5	14.4 ± 3.0
LEIDS-R total score (mean ± SD)	44.3 ± 15.5	38.1 ± 16.4
LEIDS-R HOP (mean ± SD)	5.2 ± 3.8	4.5 ± 3.8
LEIDS-R ACC (mean ± SD)	3.0 ± 2.3	2.5 ± 2.8
LEIDS-R AGG (mean ± SD)	7.7 ± 4.8	6.5 ± 4.1
LEIDS-R CTL (mean ± SD)	7.3 ± 4.0	6.7 ± 3.5
LEIDS-R RAV (mean ± SD)	9.4 ± 4.1	7.9 ± 4.0
LEIDS-R RUM (mean ± SD)	11.7 ± 4.7	10.1 ± 4.8
Past depression (MDQ) (% yes)	28.6	26.2
Abuse (% moderate to severe on CTQ)	22.9	13.8

STAXI, Spielberger State Trait Anger Expression Inventory; LEIDS-R, Leiden Index of Depression Sensitivity – Revised; HOP, Hopelessness; ACC, Acceptance; AGG, Aggression; CTL, Control; RAV, Risk Aversion; RUM, Rumination; MDQ, Mood Disorder Questionnaire; CTQ, Childhood Trauma Questionnaire.

#### Demographics

The group of high-allele carriers (MAOA-H/HH) comprises significantly more women than the low-allele (MAOA-L/LL) group (χ^2^(1) = 10.23; *P* ≤ 0.01). The groups did not differ in age.

#### STAXI

##### Main effects

No effect of genotype was observed on the STAXI scales. Women scored approximately 2 points higher than men on the STAXI Trait (*F*(1, 268) = 5.36; *P* = 0.02, partial η^2^ = 0.02) and 1 point on the STAXI State (*F*(1, 268) = 5.24; *P* = 0.02, partial η^2^ = 0.02).

##### Interaction effects

No significant interaction effect of genotype with childhood trauma and/or sex on STAXI scores was found.

#### LEIDS-R

##### Main effects

Only on the LEIDS-R HOP scale did we find a main effect. Those who had experienced moderate-to-severe levels of childhood trauma scored significantly higher (*F*(1, 268) = 4.90; *P* = 0.03, partial η^2^ = 0.02) on HOP reactivity. Main effects of MAOA genotype and sex were not significant for this scale.

##### Interaction effects

**Figure 1:**
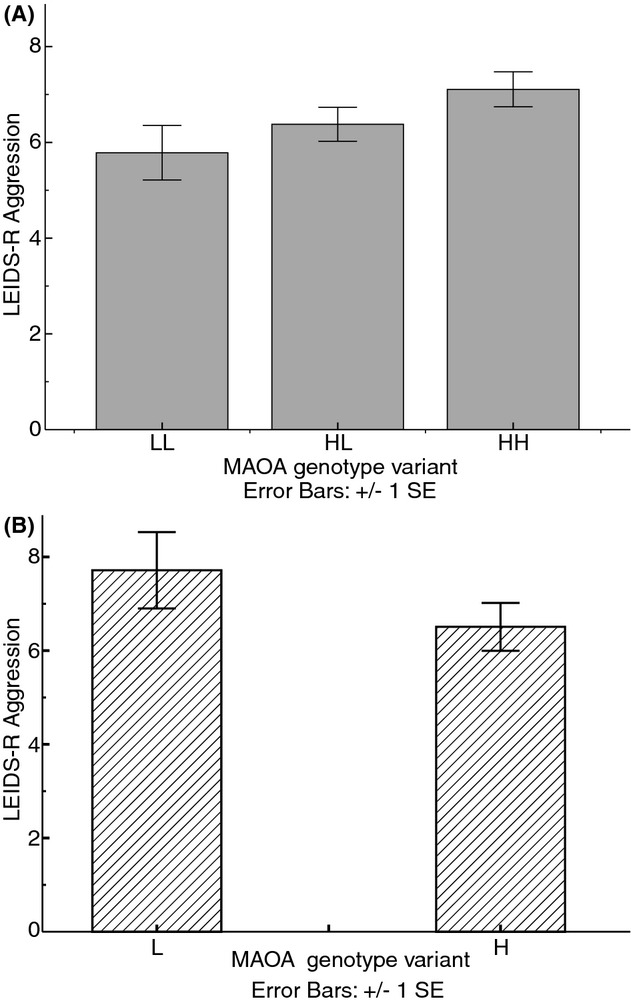
Leiden Index of Depression Sensitivity – Revised (LEIDS-R) aggression scores as a function of sex and genotype. (A) Women; (B) men. Data represent mean scores ± SE.

An interaction effect of MAOA genotype by sex was found for the AGG reactivity scale (*F*(1, 268) = 5.48; *P* = 0.02, partial η^2^ = 0.02) (Fig. 1). The secondary analyses for men and women separately revealed that women with the high-expression variant had higher scores on this subscale compared with women with the low-expression variant (*F*(1, 172) = 5.02, *P* = 0.03, partial η^2^ = 0.03). No differences were observed in men, nor were there any interactions with childhood trauma.

For the LEIDS-R total score, we found a significant interaction effect between MAOA genotype and sex (*F*(1, 268) = 7.90; *P* = 0.01, partial η^2^ = 0.03). A rerun of the analysis for men and women separately showed that MAOA-HH women had higher LEIDS-R total scores than MAOA-LL women (*F*(1, 172) = 7.06, *P* = 0.01, partial η^2^ = 0.04), while no differences were observed in men. This post hoc analysis for women separately also revealed a significant interaction between genotype and childhood trauma (*F*(1, 172) = 4.70, *P* = 0.03, partial η^2^ = 0.03). Within the group of women reporting childhood trauma, the HH carriers had higher LEIDS-R total scores compared with the LL carriers (*F*(1, 32) = 8.42, *P* = 0.01, partial η^2^ = 0.21). This effect was absent in women without a history of childhood trauma or men.

Analyses of the secondary outcome measures on the LEIDS-R showed gene by sex interactions on both RUM (*F*(1, 268) = 5.43, *P* = 0.02, partial η^2^ = 0.02) and RAV (*F*(1, 27) = 10.03, *P* ≤ 0.01, partial η^2^ = 0.04) reactivity. MAOA-HH women scored higher than MAOA-LL women on the RUM subscale. A rerun of the analyses for men and women separately showed that women with the high-expression variant scored significantly higher than those with the low-expression variant on RUM (*F*(1, 172) = 6.43, *P* = 0.01, partial η^2^ = 0.04) as well as RAV (*F*(1, 172) = 4.25, *P* = 0.04, partial η^2^ = 0.02), whereas in men no such difference was seen. A three-way interaction effect of MAOA genotype by sex by childhood trauma was detected for the RAV subscale (*F*(1, 268) = 4.67, *P* = 0.03, partial η^2^ = 0.02). A subsequent analysis for men and women with and without childhood trauma history showed that MAOA-HH women with a history of childhood trauma had higher risk aversion scores than MAOA-LL women with a history of childhood trauma (*F*(1, 32) = 5.80, *P* = 0.02, partial η^2^ = 0.15). Such effects were not observed for women without childhood trauma, neither were any main or interaction effects observed in men only.

#### MAOA genotype in women

Given the sex by genotype interactions on the LEIDS-R AGG reactivity scale, total score as well as the RUM and RAV scale, a separate analysis in women was conducted including the heterozygotes to study these interaction effects in detail.

##### Main effects

We found a significant main effect of MAOA genotype for the LEIDS-R total score (*F*(1, 326) = 3.17; *P* = 0.04, partial η^2^ = 0.02) and visual inspection suggested a dose–effect relationship. Subsequent post hoc Tukey's tests did not reveal significant group differences between the HH, HL, and LL group, but women with the HH genotype tended to have higher LEIDS-R total scores than women with the LL genotype (*P* = 0.099).

An analysis at the allele level (LL, HL vs. HH) showed a trend toward greater aggression reactivity scores in women who were homozygous for the H allele compared with those with one or two L alleles (*F*(1, 328) = 3.40, *P* = 0.07, partial η^2^ = 0.010). Such effects were not observed on the other primary outcome measures. Analyses of the secondary outcome measures showed a significant difference between genotypes on the RAV reactivity subscale (*F*(1, 326) = 3.20; *P* = 0.04, partial η^2^ = 0.01), although the post hoc group comparisons were not significant. No other effects of genotype were found.

##### Interaction effects

No interaction effects were found.

## Discussion

The aim of this study was to investigate the role of the MAOA gene and its interaction with childhood trauma and sex on measures of trait and state-dependent aggression-related behaviors in a healthy young adult sample.

We found that women with the MAOA-HH genotype scored higher on some measures of aggression compared with MAOA-LL women. Specifically, MAOA-HH women reported more aggressive thoughts and behavior in relation to sad mood (LEIDS-R AGG scale) compared with MAOA-LL women. Such effects on the LEIDS-R AGG scale did not occur in men, nor did we see any effects on more general trait and state measures of aggressive behaviors such as the STAXI. This discrepancy between the results on the LEIDS-R and the STAXI may be explained by the fact that the STAXI contains two separate scales for state and for trait, whereas the LEIDS-R measures aggression in the context of dysphoria.

The notion that the effects of MAOA genotype may be context dependent is consistent with an experimental study in healthy males (McDermott et al. 2009). Using an aggression provocation task, it was found that the impact of the MAOA-L variant on aggressive behavior in males was largest in the context of aggression provocation (McDermott et al. 2009).

The presently found sex-specific effects and their direction are in line with Sjöberg et al. (2007), who reported more criminal behavior in MAOA-HH adolescent girls with higher psychosocial risk compared with adolescent girls without this risk. Our study is a first in showing an association between the high-expression MAOA variant and aggression-related behaviors in adult women. Sjöberg et al. found only effects in girls with higher levels of psychosocial adversity, whereas in our sample, the effects were irrespective of childhood trauma history. Differences in the type of childhood trauma measured (Sjöberg: multifamily housing and sexual abuse; current study: emotional and physical neglect and abuse, sexual abuse) may account for the discrepancies in findings between the studies.

We also found sex-specific effects of the MAOA-H variant on total LEIDS-R score, RAV and RUM. RUM is known to predict higher levels of depressive symptoms, recurrence of depressive episodes, as well as chronicity (Nolen-Hoeksema 1991; Robinson and Alloy 2003). Antypa et al. (2010) found higher scores on the LEIDS-R total score as well as on the RUM scale in healthy individuals who are carriers of the s allele of the serotonin transporter promoter polymorphism, a genotype commonly associated with depression (Du et al. 1999; Kaufman et al. 2006).

We observed one significant three-way interaction of sex, genotype, and childhood trauma on the LEIDS-R RAV scale. Specifically, an association between risk aversion scores and the high MAOA expression variant was found only in women with a history of childhood trauma. The RAV scale measures the tendency to avoid not only risk but also interpersonal conflict and is the opposite of aggression. As the HH variant of the MAOA genotype is associated with increased aggression, we may speculate that the observed association between the MAOA-HH variant and risk aversion suggests that in the context of an early adversity, increased risk aversion behavior in HH homozygotes may be a compensatory mechanism for increased feelings of aggression. Another explanation of increased aggression in combination with increased risk aversion in the context of early adversity is that MAOA-HH girls who show more aggression during early childhood may have experienced increased punishment for their aggression by their parents or caretakers, thus learning to avoid certain behaviors to avoid punishment or abuse. However, we did not have sufficient information to test for possible mechanisms accounting for these effects. Individuals who had experienced trauma in childhood had higher HOP reactivity scores than individuals without any history of childhood trauma, irrespective of sex or genotype. Interestingly, HOP reactivity has been found to be a predictor of risk for suicidal ideation or attempt in formerly and currently depressed samples (Williams et al. 2008; Antypa et al. 2010). In addition, childhood trauma has been shown to be a predictor of suicidality (Beautrais et al. 1996; Bernet and Stein 1999; Johnson et al. 1999; Dube et al. 2001; Heim and Nemeroff 2001; Agerbo et al. 2002; Brent et al. 2002). Since our sample comprises healthy individuals, this study extends these observations, suggesting that childhood trauma may set the stage for tendencies toward thoughts of hopelessness. This might in turn lead to suicidal ideation, especially in the context of further genetic susceptibility or further stressors.

The current study has some limitations, one of them being the relatively small number of men in the sample. Therefore, we cannot rule out the possibility that the lack of effects in men is due to a type II error. Indeed, Williams et al. (2009) found in a healthy sample that MAOA-L men had higher antisocial trait scores than men with the MAOA-H genotype, while no such difference was found in women. Notably, the majority of Williams' et al. sample consisted of men (67%). In interpreting our results, we should thus consider the possibility that the lack of results in men in the current sample may be due to its smaller size. Furthermore, we did not correct for multiple testing. Hence, the risk of false-positive observations cannot be ruled out. However, our study had the a priori aim to compare different types of aggression measures and their relationship to the MAOA genotype.

Another limitation is that our aggression measures were all based on self-report. It would be of interest to extend this study using other measures of aggression such as observational measures, diary techniques, or laboratory aggression-induction procedures.

A strength of this study is that we recruited a sample that was relatively homogeneous in terms of age, education, and ethnicity. Furthermore, participants were screened for mental health problems before enrolling in the study. However, a disadvantage of our recruitment strategy is that university students are likely to score relatively low on violence and aggression compared with the general population. Although mean scores on the STAXI (both State and Trait) did not differ much from norm scores for this questionnaire, it would be of interest for future studies to use the same methods and procedures in a community sample.

To summarize, this study showed that some of the associations between aggression, genes, and diagnosis previously observed in nonadult patient samples can be generalized to healthy young adult samples. This is reflected by elevated scores on assessments measuring the tendency to display aggressive behaviors/thoughts in a context of sad mood, rather than in behavior or disease pattern itself.
