# The effects of MAOA genotype, childhood trauma, and sex on trait and state-dependent aggression

**DOI:** 10.1002/brb3.119

**Published:** 2013-01-09

**Authors:** Floor E A Verhoeven, Linda Booij, Anne-Wil Kruijt, Hilâl Cerit, Niki Antypa, Willem Van der Does

*Brain and Behavior* 2012; **2**(6): 806–813

On page 807, we made some mistakes in the description of the inclusion and exclusion criteria. Specifically,

- we did not exclude smokers;
- we did not ask participants about medication use;
- the age range was not 18–35 years, but 17–39 years.

We regret these errors. They have no consequences for the reported analyses and conclusions.
